# Role of Transcription Factor, LIM Homeobox 9 (LHX9) in Inflammatory Response by PGE2 and Thrombin in SERPINA1‐Silencing Endometrial Stromal Cells

**DOI:** 10.1002/mrd.70046

**Published:** 2025-08-13

**Authors:** Kazuya Kusama, Misuzu Ashihara, Moana Okita, Kanoko Yoshida, Masumi Suzuki, Kaito Suzuki, Rena Hosokawa, Mikihiro Yoshie, Junya Kojima, Yumi Mizuno, Masanori Ono, Hirotaka Nishi, Takeshi Kajihara, Kazuhiro Tamura

**Affiliations:** ^1^ Department of Endocrine Pharmacology Tokyo University of Pharmacy and Life Sciences Tokyo Japan; ^2^ Department of Obstetrics and Gynecology Tokyo Medical University Tokyo Japan; ^3^ Department of Obstetrics and Gynecology Saitama Medical University Saitama Japan

**Keywords:** endometrial stromal cell, endometriosis, LHX9, prostaglandin E_2_, SERPINA1, thrombin

## Abstract

Endometriosis is hypothesized to result from retrograde menstruation where cell debris including endometrial stromal cells (ESCs) travel through the fallopian tubes. This chronic inflammatory disease is characterized by inflammatory and fibrotic endometrial tissue. We have previously observed reduced expression of the anti‐inflammatory factor SERPINA1 in endometriosis‐like lesions in a mouse model implanted with human ESCs. Additionally, pro‐inflammatory factors present in peritoneal hemorrhage exacerbated inflammation in these grafts, partly through prostaglandin (PG) E2 and thrombin. However, it remains unclear whether the reduction of SERPINA1, in combination with PGE2 and thrombin, synergistically influences the expression of inflammatory factors in endometriosis lesions and the underlying mechanisms. We analyzed RNA sequencing data from ESCs treated with SERPINA1 siRNA and PGE2/thrombin, comparing them to data sets derived from ESCs subjected to either SERPINA1 knockdown or PGE2/thrombin treatment. Comparative analysis identified 49 transcripts that were upregulated under both conditions and enriched for transcription regulatory genes, including SNAI1, HDAC5, PBX1, SOX4, EPAS1, LHX9, and MAFK. Silencing SNAI1, HDAC5, SOX4, EPAS1, or LHX9 suppressed IL6, CXCL8, and IL1B expression, which had been upregulated by SERPINA1 siRNA and PGE2/thrombin. Among these genes, LHX9 expression was significantly elevated in ectopic lesions, predominantly localized to stromal and glandular epithelial cells, with more pronounced expression during the secretory phase. LHX9 levels were also increased in endometriotic lesions compared to the normal endometrium. In conclusion, reduced SERPINA1 expression in ectopic ESCs, combined with PGE2/thrombin, induces inflammatory cytokine expression linked to LHX9. Pharmacological targeting of LHX9 may present a promising therapeutic strategy for mitigating chronic inflammation in endometriotic lesions.

## 1 Introduction

Endometriosis is a chronic inflammatory disease characterized by the presence of inflamed and fibrotic endometrial tissue outside the uterine cavity, affecting sites such as the ovaries and peritoneum (Bulun et al. 2019; Giudice and Kao 2004; Pullen et al. 2011; Zondervan et al. 2018). This condition may arise from retrograde menstrual flow, in which endometrial stromal and epithelial cells travel backward through the fallopian tubes into the pelvic cavity (Bulun et al. 2019; Giudice and Kao 2004). Within ectopic lesions, chronic inflammation promotes epithelial‐mesenchymal transition and fibrosis, contributing to the progression of endometriosis (Zondervan et al. 2018). However, given that menstrual blood reflux is reported in 90% of women of reproductive age, the precise causal relationship remains ambiguous.

Our experimental endometriosis‐like mouse model, in which human endometrial stromal cells (ESCs) and glands were used to create lesions adjacent to the surgical site of ovariectomy, previously demonstrated that hemorrhage due to ovariectomy facilitates the progression of endometriosis‐like lesions. In this model, levels of interleukin 6 (IL6) and prostaglandin E2 (PGE_2_) were elevated, while levels of the serine protease inhibitor SERPINA1 were reduced in endometriosis‐like lesions (Shinohara et al. 2012; Tamura et al. 2015). Protease‐activated receptors (PARs) activation may contribute to the pathogenesis of endometriosis (Osuga et al. 2008). Treatment of endometrial stromal and glandular epithelial cells with a PAR1 agonist thrombin and PGE_2_ present in menstrual blood, significantly increases IL6 secretion, paralleling observation in endometriosis lesions (Kusama et al. 2021a, b; Shinohara et al. 2012). Notably, serum concentrations of IL6 and IL8 in endometriosis patients are higher than in healthy women (Othman et al. 2008; Sikora et al. 2017), and PGE_2_ production is also elevated in these patients (De Leon et al. 1988; Liu et al. 2011; Lucidi et al. 2005; Maybin et al. 2011).

SERPINA1 is broadly expressed in various cell types and localized to the cytoplasm and endoplasmic reticulum. Previous studies have demonstrated that decreased intracellular SERPINA1 levels exacerbate the inflammatory response in endometriosis‐like lesions in mice (Tamura et al. 2015) and heighten ER stress‐induced cytokine production in cultured human adipocytes and trophoblast cells, given the role of SERPINA1 in protecting cells from inflammatory factors (Ando et al. 2021; Yoshida et al. 2021). Interestingly, SERPINA1 knockdown upregulates toll‑like receptor (TLR) 3 and TLR4 in ESCs (Kusama et al. 2022). Conversely, purified SERPINA1 treatment suppresses inflammatory cytokine production in vitro (Tamura et al. 2015) and improves survival in mice with peritonitis or sepsis (Kaner et al. 2015). Several studies have reported that SERPINA1 mitigates organ damage in a serine protease activity‐independent manner (Gou et al. 2021; Jonigk et al. 2013; Pott et al. 2009; Yoshida et al. 2021, 2022). However, the intricate molecular mechanisms by which SERPINA1, PGE2, and thrombin influence the expression of inflammatory factors associated with the development of endometriosis lesions are not fully elucidated. This study aimed to identify novel molecules implicated in inflammation through RNA sequencing of ESCs subjected to SERPINA1 knockdown, treated with PGE_2_/thrombin, exploring the signaling for the synergistic effects of SERPINA1 reduction and PGE_2_/thrombin in ESCs.

## 2 Materials and Methods

### 2.1 Endometrial Tissues Collection

To isolate endometrial stromal cells, eutopic endometrial tissues in the proliferative phase were collected from patients with endometrioma (*n *= 3). Additionally, for gene expression analyses, both eutopic and ectopic endometrial tissues were collected in pairs from the same patients with ovarian endometriosis (*n *= 7) (Figure 4A). For immunofluorescence analyses, endometrial eutopic tissues were collected via surgical curettage from other patients with endometrioma (*n *= 6) undergoing surgery for ovarian endometriosis. The samples consisted of endometrial tissues that had been scraped rather than whole‐layer specimen collection and included samples from the proliferative (*n *= 3) and secretory (*n *= 3) phases. Furthermore, ectopic endometriotic tissue samples (*n *= 3) were obtained exclusively from patients with endometriosis (Figure 4B,C). All patients were under 45 years of age and had regular menstrual cycles of 28–32 days. Menstrual phase was determined based on detailed menstrual histories recorded over at least the preceding 6 months. All participants (*n* = 19) provided written informed consent before enrollment. The study was conducted in accordance with the Declaration of Helsinki and was approved by the Clinical Research Ethics Committees of Tokyo Medical University Hospital (approval number: 2017086, T2022‐0248), Saitama Medical University (approval number: 18085), and Tokyo University of Pharmacy and Life Sciences (approval number: 15‐11, 15‐15, W2023‐001).

### 2.2 Endometrial Stromal Cells (ESCs) Culture and Treatment Conditions

Endometrial tissues from the proliferative phase were washed with Ca^2+^/Mg^2+^‐free Hank's balanced salt solution (Fujifilm Wako Pure Chemical Corp., Osaka, Japan), cut into small pieces, and then digested for 2 h at 37°C in Ca^2+/^Mg^2+^‐free PBS containing type I collagenase (2.5 mg/mL; Sigma‐Aldrich, Tokyo, Japan), DNase I (25 µg/mL; Nippon Gene, Tokyo, Japan), and PSN (100 µg/mL penicillin, 100 µg/mL streptomycin, and 200 µg/mL neomycin; Thermo Fisher Scientific, Waltham, MA, USA). Primary cultures of endometrial cells were prepared as previously described (Kusama et al. 2022). Primary ESCs were resuspended in Dulbecco's modified Eagle's medium/F12 (DMEM/F12, 1:1; Fujifilm Wako Pure Chemical Corp.) supplemented with 10% fetal bovine serum, antibiotics, and antimycotics. The ESCs were seeded onto culture dishes and cultured at 37°C in humidified air containing 5% CO_2_. ESCs grown to 50% confluency in 24‐well plates were transfected siRNA or short hairpin (sh)RNA, and then the medium was removed, and cells were cultured for 24 h with or without prostaglandin E2 (PGE2; 1 µM, Fujifilm Wako Pure Chemical Corp.) and thrombin (10 U/mL, Fujifilm Wako Pure Chemical).

### 2.3 Transfection of Small Interfering (Si)RNA and the Expression Plasmid Construct

ESCs were transfected with either a nontargeting control siRNA or with SERPINA1 siRNA (EHU090971, Sigma‐Aldrich) using Lipofectamine RNAiMAX (Thermo Fisher Scientific) (Yoshida et al. 2022). The SERPINA1 expression vector pTCP (BC011991) was purchased from TransOMIC Technologies (Huntsville, AL, USA). The pTCP‐SERPINA1 plasmid (1 μg) was transfected into ESCs by electroporation using the Neon transfection system (Thermo Fisher Scientific), according to the manufacturer's instructions. Cells were pulsed twice with 1400 V for 20 ms, and transfected cells were selected using puromycin (3 μg/mL) for 48 h (Yoshida et al. 2021).

### 2.4 RNA Extraction and Quantitative RT‐PCR (qPCR)

Total RNA was extracted from endometriosis tissue and cultured cells using Isogen II (Nippon Gene), according to the manufacturer's instructions. Reverse transcription was performed using a ReverTra Ace qPCR RT Kit (Toyobo, Osaka, Japan). Synthesized cDNA was then subjected to qPCR amplification using PowerUp SYBR Green Master Mix or PowerTrack SYBR Green Master Mix (Thermo Fisher Scientific). Alternatively, PCR analysis was conducted using the Luna Universal One‐Step RT‐qPCR Kit (New England Biolabs Japan Inc.), which enables simultaneous reverse transcription and amplification. The primers are listed in Table 1. Calibration curves were generated to assess the amplification efficiency for each target gene and the reference gene glyceraldehyde‐3‐phosphate dehydrogenase (*GAPDH*), demonstrating comparable efficiency. Ct values were determined using Sequence Detection System software v2.3 (Thermo Fisher Scientific) (Kusama et al. 2021a).

**Table 1:** Sequence of primers used for real‐time quantitative PCR.

Name (accession no.)	Sequence (5′—3′)	Product length (bp)
*GAPDH* NM_002046.7	AGCCACATCGCTCAGACA	66
	GCCCAATACGACCAAATCC	
*IL1B* NM_000576.3	TGATGGCTTATTACAGTGGCAATG	131
	GTAGTGGTGGTCGGAGATTCG	
*IL6* NM_000600.5	CAGGAGCCCAGCTATGAACT	85
	AGCAGGCAACACCAGGAG	
*CXCL8* NM_000584.4	AAGCATACTCCAAACCTTTCCA	123
	CCAGACAGAGCTCTCTTCCA	
*SNAI1* NM_005985.4	ACTGCAACAAGGAATACCTCAG	244
	TGGCACTGGTACTTCTTGAC	
*HDAC5*	CGCTGAGAATGGCTTTACTGGC	108
NM_005474.5	GTGTAGAGGCTGAACTGGTTGG	
*PBX1*	GGAGGATACAGTGATGGACTCG	144
NM_002585.4	GGAGGTATCAGAGTGAACACTGC	
*SOX4*	GACATGCACAACGCCGAGATCT	123
NM_003107.3	GTAGTCAGCCATGTGCTTGAGG	
*EPAS1* NM_001430.5	CTGTGTCTGAGAAGAGTAACTTCC TTGCCATAGGCTGAGGACTCCT	145
*LHX9* NM_020204.3	ACCTGCTTTGCCAAGGACGGTA TGACCATCTCCGAGGCGGAAAT	112
*MAFK* NM_002360.4	CTGCGCTCCAAGTACGAGGCG TCGGTGGACTTGACGATGGTGA	113
*SERPINA1*	TCAAGGAGCTTGACAGAGACAC	94
NM_001127704.2	TCGGTGTCCTTGACTTCAAAGG	

### 2.5 RNA Sequencing (RNA‐Seq), Gene Ontology (GO), and Pathway Analyses

RNA‐seq analysis was performed with RNA extracted from cultured ESCs using Isogen II (Nippon Gene). High‐throughput sequencing libraries were prepared using a TruSeq Stranded mRNA LT Sample Prep Kit (Illumina, San Diego, CA, USA), according to the manufacturer's instructions, and data analysis was performed by Macrogen Japan (Kyoto, Japan). Primary sequence data were deposited in the DDBJ (DNA Data Bank of Japan) Sequence Read Archive (https://www.ddbj.nig.ac.jp/dra/index-e.html; accession numbers: DRR304262 to DRR304273). Data analysis was performed as described previously (Kusama et al. 2021a). Briefly, trimmed sequences were analyzed using the STAR/RSEM/edgeR pipeline, the human genome (hg38), and reference annotations obtained from the UCSC genome browser (https://genome.ucsc.edu). Significantly differentially expressed genes (DEGs) were identified based on CPM (count per million) levels. GO and Enriched Signaling Pathway analyses were performed using the Enrichr tool (http://amp.pharm.mssm.edu/Enrichr/).

### 2.6 Transfection of shRNA

shRNA sequences targeting SNAI1, HDAC5, PBX1, SOX4, EPAS1, LHX9, and MAFK were designed and cloned into a linearized pLKO.1 vector by PCR using PrimeSTAR Max DNA Polymerase (Takara Bio, Shiga, Japan) and the NEBuilder HiFi DNA Assembly system (New England Biolabs Japan, Tokyo, Japan). The shRNA and primers are listed in Table 2. The control shRNA, pLKO.1‐shSCR (plasmid 1864), which does not target any known mammalian gene, was obtained from Addgene. Lentiviral particles were generated using the pLKO.1 construct containing the shRNA sequence and Lentiviral High Titer Packaging Mix (Takara Bio) in Lenti‐X 293 T cells (Takara Bio) for transduction in the shRNA‐mediated knockdown system. Culture supernatants containing lentiviral particles were harvested, centrifuged to remove cellular debris, filter sterilized, and stored at −80°C until used. ESCs were transduced with lentiviral particles and selected using 3 µg/mL puromycin dihydrochloride (Nacalai Tesque) for 2 days. Following selection, cells were cultured for an additional day without puromycin before collecting samples for mRNA analyses (Imakawa et al. 2016).

**Table 2:** Specific shRNA and primer sequences.

Target gene	Sequence (5′—3′)
shRNAs	
*SNAI1*	TAGTTATATGTACAGTTTATT
*HDAC5*	GGGGGAAATGCCTTAATTTCA
*PBX1*	GCATCAGTGCTAATGGAGGTT
*SOX4*	AGCGACAAGATCCCTTTCATT
*EPAS1*	ATGTATGTATGTATTATTATT
*LHX9*	GCTGCTTCACTTGTTCCACTT
*MAFK*	TGGTAGGTAATCCATATTGGA
Primers	
pLKO.1‐F	GGTGTTTCGTCCTTTCCACAAG
pLKO.1‐R	TCGACCTCGAGACAAATGGCA

### 2.7 Immunohistofluorescence

Paraffin‐embedded sections of endometriotic tissue were immunostained using antibodies against LHX9, following a previously described protocol (Kusama et al. 2022). Briefly, sections were rehydrated, subjected to antigen retrieval by boilling for 20 min in 10 mM citrate buffer (pH 6.0), and subsequently incubated overnight at 4°C with LHX9‐specific antibodies (1:100; Sigma‐Aldrich) or normal rabbit IgG (1:100, sc‐2027, Santa Cruz Biotechnology, Dallas, TX, USA) as a negative control. Subsequently, sections were incubated with Alexa Fluor 488‐labeled alpaca anti‐rabbit IgG (Life Technologies Corporation, Carlsbad, CA, USA). Nuclei were counterstained using 4′, 6‐diamidino‐2‐phenylindole (DAPI; Life Technologies Corporation). The fluorescently labeled cells were analyzed using a BZ‐X800 microscope (Keyence, Osaka, Japan).

### 2.8 Statistical Analysis

The qPCR data represent the results of three or more independent experiments, with each sample assayed in triplicate. Data are expressed as the mean ± SEM and were compared using Dunnett's test in R software (v4.0.5). *p* < 0.05 was considered statistically significant. In RNA‐seq analysis, a false discovery rate‐adjusted *p* value (*q*‐value) < 0.05 was considered statistically significant (Kusama et al. 2021a).

## 3 Results

### 3.1 Effect of PGE2/Thrombin and SERPINA1 on the Expression of IL6 and CXCL8

**Figure 1:**
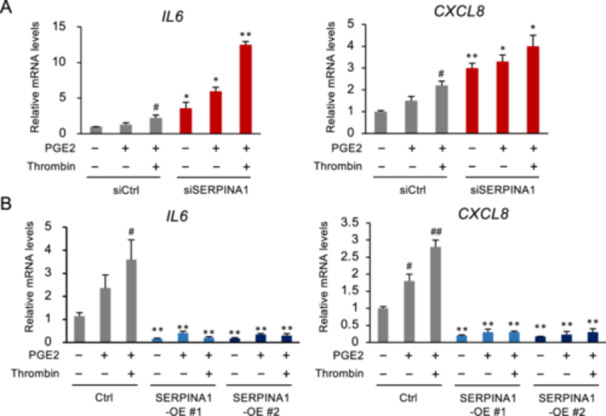
Effect of PGE2/thrombin on the expression of *IL6* and *CXCL8* in ESCs with SERPINA1 silencing or overexpression. (A) ESCs were transfected with siRNA specific for SERPINA1 (siSERPINA1) for 24 h, followed by treatment with PGE2 (1 µM) and thrombin (10 U/mL) for 6 h. The levels of *IL6* and *CXCL8* expression were determined using qPCR, with *GAPDH* as the reference gene. Data are presented as mean ± SEM from three independent experiments, each performed in duplicate. ^#^ *p* < 0.05 vs. siCtrl‐intact. *p < 0.05, **p < 0.01 vs. control (Ctrl) for each treatment group. (B) ESCs overexpressing SERPINA1 (SERPINA1‐OE) were treated with PGE2 (1 µM) and thrombin (10 U/mL) for 6 h. Expression levels of *IL6* and *CXCL8* were determined using qPCR, with *GAPDH* as the reference gene. Data are expressed as mean ± SEM from three independent experiments, each performed in duplicate. ^#^ *p* < 0.05, ^##^ *p* < 0.01 vs. siCtrl‐intact. **p < 0.01 vs. Ctrl‐each treatment group.

To assess the impact of SERPINA1 knockdown and PGE2/thrombin on the expression of *IL6* and *CXCL8* in ESCs, we conducted experiments using ESCs. Knockdown of SERPINA1 resulted in a significant upregulation of *IL6* and *CXCL8* expression (Figure 1A). Exposure to PGE2 and thrombin further enhanced *IL6* and *CXCL8* expression levels (Figure 1A). To further investigate the role of SERPINA1 in the expression of inflammatory cytokines, ESCs were transfected with a SERPINA1 overexpression vector. Conversely, SERPINA1 overexpression led to a marked reduction in *IL6* and *CXCL8* expression levels, which remained unchanged despite PGE2 or thrombin treatment (Figure 1B).

### 3.2 RNA‐Seq Analysis of PGE2/Thrombin‐Treated ESCs With SERPINA1 Knockdown

**Figure 2:**
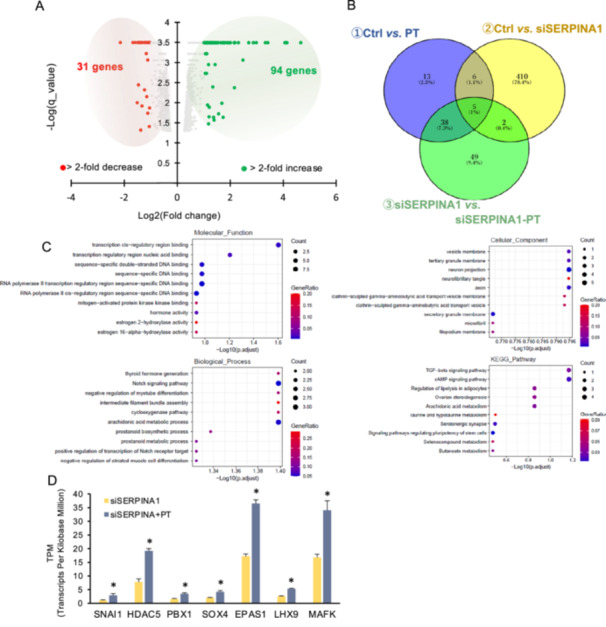
RNA‐seq analysis of SERPINA1‐knockdowned ESCs after PGE2/thrombin treatment. ESCs were transfected for 24 h with siRNA specific for SERPINA1 (siSERPINA1) and then treated with PGE2 (1 µM) and thrombin (10 U/mL) (PT) for 6 h. RNA was extracted and subjected to RNA sequencing. (A) Volcano plot showing the expression of transcripts identified by RNA‐seq. The transcripts highlighted in red or green were twofold differentially expressed (*q* value < 0.05). (B) Venn diagram of the group of factors that are altered by PGE2 and thrombin treatment only under conditions of suppressed SERPINA1 expression, analyzed in comparison with previous transcriptome data. (C) Differentially expressed genes with more than twofold upregulation were functionally classified using Gene Ontology (GO) analyses of biological processes, cellular components, molecular functions, and KEGG pathway data sets. (D) Differential expression of genes *SNAI1*, *HDAC5*, *PBX1*, *SOX4*, *EPAS1*, *LHX9*, and *MAFK*, identified by GO molecular function database analysis with RNA‐seq data. *p < 0.01 vs. siSERPINA1.

To further elucidate the molecular mechanism underlying the upregulation of inflammatory cytokine expression by SERPINA1 knockdown and PGE2/thrombin, RNA‐seq analysis was performed in ESCs treated with SERPINA1 siRNA and PGE2/thrombin. RNA‐seq analysis identified 125 differentially expressed genes (DEGs), including 94 upregulated and 31 downregulated genes, relative to ESCs treated with SERPINA1 siRNA alone (Figure 2A). To assess the potential synergistic effects of SERPINA1 knockdown and PGE2/thrombin treatment in ESCs, data sets from ESCs subjected to either SERPINA1 knockdown, PGE2/thrombin treatment, or both were compared. Comparative analysis showed 49 transcripts as genes upregulated by both SERPINA1 siRNA and PGE2/thrombin treatments (Figure 2B). KEGG pathway and GO enrichment analyses revealed enrichment in genes associated with “Transcription cis‐regulatory region”, “TGF‐beta signaling pathway”, and “cAMP signaling pathway” (Figure 2C). Genes related to transcription cis‐regulatory regions included Snail family transcriptional repressor 1 (SNAI1), histone deacetylase 5 (HDAC5), PBX homeobox 1 (PBX1), SRY‐box transcription factor 4 (SOX4), endothelial PAS domain protein 1 (EPAS1), LIM homeobox 9 (LHX9), and MAF bZIP transcription factor K (MAFK) (Figure 2D).

### 3.3 Functional Analysis of Identified DNA‐Binding Factors in the Expression of Inflammatory Cytokines

**Figure 3:**
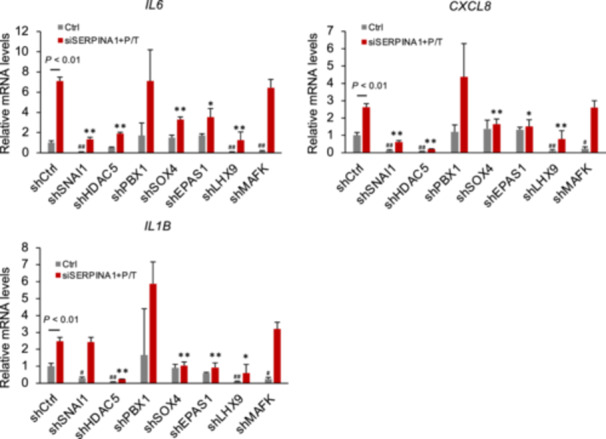
Effects of knockdown of identified DNA‐binding factors on inflammatory cytokine expression. ESCs pre‐transfected with shRNAs targeting SNAI1, HDAC5, PBX1, SOX4, EPAS1, LHX9, or MAFK, followed by treatment with SERPINA1 siRNA (siSERPINA1) for 24 h, and subsequently PGE2 (1 μM) and thrombin (10 U/mL) (PT) for 18 h. Expression of *IL6*, *CXCL8*, and *IL1B* in ESCs was determined using qPCR, with *GAPDH* serving as the reference gene. Data are presented as the mean ± SEM from three independent experiments, each performed in duplicate. *p < 0.05, **p < 0.01 vs. shCtrl‐siSERPINA1, PGE2, and thrombin. ^#^ *p* < 0.05, ^##^ *p* < 0.01 vs. shCtrl‐Ctrl.

To investigate whether transcriptionally regulated region‐associated genes are involved in inflammatory cytokine expression in SERPINA1‐knockdown ESCs treated with PGE2/thrombin, cells were transfected with shRNA vectors specific to these genes. Silencing HDAC5, SOX4, EPAS1, or LHX9 inhibited the expression of *IL6*, *CXCL8*, and *IL1B*, which was upregulated by SERPINA1 siRNA and PGE2/thrombin treatment (Figure 3). SNAI1 shRNA had no effect on IL1B expression. Moreover, shRNAs targeting MAFK and PBX1 did not influence the expression of these cytokines. Furthermore, the suppression of LHX9 and SNAI1 expression also reduced the basal levels of cytokine expression.

### 3.4 LHX9 Expression and Localization in the Endometriotic Lesions

**Figure 4:**
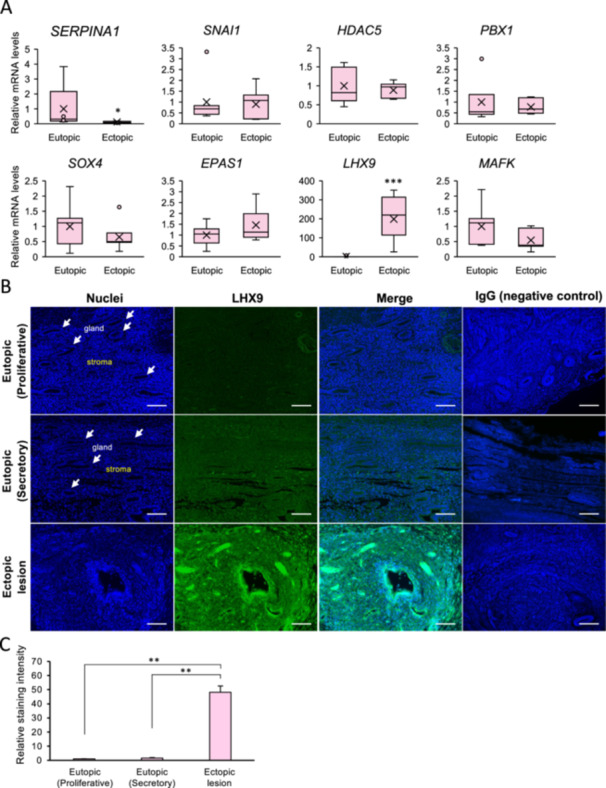
LHX9 expression and localization in endometriotic lesion. (A) The expression levels of *SERPINA1*, *SNAI1*, *HDAC5*, *PBX1*, *SOX4*, *EPAS1*, *LHX9*, and *MAFK* were quantified by qPCR in eutopic and ectopic endometrial tissues from seven patients with endometriosis using qPCR, with *GAPDH* as the reference gene. Values are presented as mean ± SEM. *p < 0.05, ***p < 0.001 vs. Eutopic. (B) For immunofluorescence analysis of LHX9, sections of normal endometrium tissues in the proliferative (*n* = 3) and secretory (*n* = 3) phases, as well as ectopic endometriotic tissues (*n* = 3), were immunostained for LHX9, followed by nuclear counterstaining with DAPI. Arrows indicate the endometrial gland. Scale bar = 100 μm. (C) Relative staining intensity was calculated and presented as ratios normalized to the proliferative phase. **p < 0.01.

To determine whether identified transcriptionally regulated region‐associated genes are expressed and altered in endometriosis lesions, we performed qPCR and immunohistochemical analysis using ectopic lesions and eutopic endometrial tissues from patients with endometriosis. Consistent with previous studies using model mice (Tamura et al. 2015), *SERPINA1* expression was decreased in endometriotic lesions compared to eutopic endometrium from patients. In contrast, *LHX9* expression was increased in ectopic lesions. However, the expression levels of *SNAI1*, *HDAC5*, *PBX1*, *SOX4*, *EPAS1*, and *MAFK* did not show significant increases in endometriotic lesions (Figure 4A). Next, the expression and localization of LHX9 were characterized in the endometrium during proliferative and secretory phases as well as in endometriosis lesions using immunohistochemical staining (Figure 4B). LHX9 was localized to stromal and glandular epithelial cells. Its expression levels in the secretory phase were higher than those in the proliferative phase. Furthermore, compared to normal endometrium, LHX9 expression was further increased in endometriotic lesions (Figure 4C).

## 4 Discussion

This study demonstrated that PGE2 or thrombin stimulated the expression of inflammatory cytokines IL6 and CXCL8. This effect was further amplified by SERPINA1 knockdown and, conversely, suppressed by SERPINA1 overexpression. Global transcriptome analysis of ESCs with SERPINA1 silencing and PGE2 and thrombin treatment revealed significant changes in gene expression. Comparative analysis of ESCs data sets with SERPINA1 knockdown and/or PGE2/thrombin treatment identified 49 genes that were upregulated by both SERPINA1 siRNA and PGE2/thrombin. Enrichment analyses of upregulated DEGs showed that SERPINA1 knockdown and PGE2/thrombin influenced genes related to “Transcription cis‐regulatory region”‐related genes, including SNAI1, HDAC5, PBX1, SOX4, EPAS1, LHX9, and MAFK. To further assess the role of these regulatory factors, we examined their effects on the expression of inflammatory cytokines in ESCs treated with SERPINA1 siRNA and PGE2/thrombin. HDAC5, SOX4, EPAS1, LHX9, and SNAI1 shRNAs inhibited the expression of *IL6*, *CXCL8*, and *IL1B* upregulated by SERPINA1 siRNA and PGE2/thrombin, although PBX1 and MAFK shRNA did not affect those cytokine expressions. Moreover, LHX9 expression was significantly elevated in ectopic endometrium compared with eutopic endometrium in patients with endometriosis. These findings suggest that the combined effects of PGE2/thrombin treatment and SERPINA1 knockdown drive inflammatory cytokine production through the activation of the DNA‐binding genes LHX9.

LHX9 belongs to the member of the LIM‐homeodomain (LIM‐HD) family of genes, which plays a crucial role in various functions during embryonic development (Singh et al. 2022). The activity of LIM‐HD proteins is modulated by co‐regulators. Lhx9 facilitates the differentiation of specific neural cell types and regulates gene expression essential for brain function development. In mice, Lhx9 is essential for the somatic cell proliferation in genital ridges and plays a role in regulating testosterone production by Leydig cells. Lhx9 transcripts are detected in the urogenital ridges of mice at embryonic day 9.5 and subsequently localize to the interstitial region following morphological differentiation (Birk et al. 2000). The knockdown of LHX9 suppressed the apoptosis pathway, Hippo signal pathway, and p53 signaling pathway in hematopoietic cells (Zhou et al. 2018). Our results indicate that LHX9 knockdown decreases the expression of inflammatory cytokines, and LHX9 is significantly upregulated in endometriotic lesions. These findings suggest that LHX9 may contribute to inflammatory cytokine expression and the regulation of the proliferation and differentiation of eutopic endometrial stromal cells under conditions mimicking a pathological environment. Further studies are required to elucidate the molecular mechanisms through which this DNA‐binding factor regulates the inflammatory cytokine expression in ESCs.

Fibrogenesis and fibroproliferation are critical processes stages in the formation of endometriotic lesions and are strongly linked to severe pain. The presence of myofibroblasts in endometriotic lesions may be involved in the pathogenesis of the disease (Anaf 2000; Barcena de Arellano et al. 2011; Zhang et al. 2016). Myofibroblasts arise from the transformation of various cell lineages, including tissue‐resident fibroblasts and epithelial cells, via epithelial‐to‐mesenchymal transition (EMT) (Vigano et al. 2018). The EMT of endometrial cells plays a key role in the pathophysiology of endometriosis and is driven by the TGF‐β and Wnt signaling pathways (Yang and Yang 2017). In this study, the enrichment pathway analyses of upregulated DEGs revealed that SERPINA1 knockdown and PGE2/thrombin treatment activated TGF‐β signaling pathway. Previous studies have demonstrated that PGE2/thrombin activated activin A, a TGF‐β superfamily factor production, which subsequently EMT induces and promotes connective tissue growth factor (CTGF) expression in ESCs (Kusama et al. 2021a). CTGF serves as a fibrosis marker in endometrial diseases such as endometriosis and intrauterine adhesions (Li et al. 2016; Matsuzaki and Darcha 2014). Our previous study demonstrated that CTGF enhanced the expression of the fibrosis markers ACTA2, COL1A1, and FN1 in ESCs. In human endometriotic cells, TGF‐β signaling triggers EMT, which subsequently leads to fibroblast‐to‐myofibroblast transition (FMT) (Zhang et al. 2016). The present study is the first to demonstrate that SERPINA1 expression is significantly reduced in ectopic compared to eutopic endometrium in patients with endometriosis. Furthermore, our findings indicate that the suppression of SERPINA1 expression in eutopic endometrial stromal cells significantly upregulates inflammatory cytokines linked to endometriosis. These findings suggest that eutopic endometrial cells may undergo fibrotic changes through EMT and FMT, stimulated by inflammatory factors in menstrual fluid. Under reduced SERPINA1 conditions, PGE2/thrombin might promote EMT and FMT of ESCs, potentially exacerbating fibrosis in both eutopic ESCs and endometriotic lesions. However, the underlying cause of decreased SERPINA1 expression in these lesions remains unknown. Moreover, ESCs from the eutopic tissues used in this study were derived from patients with endometriosis, and it is unclear whether the same biological reactions occur in the corresponding tissues of healthy individuals. Our findings support the hypothesis that eutopic endometrial cells, upon reflux into the peritoneal cavity via retrograde menstruation, may undergo EMT, FMT, and fibrotic remodeling driven by pro‐inflammatory factors present in the menstrual fluid, thereby contributing to the development of endometriotic lesions.

Collectively, these results indicate that SERPINA1 inhibition, combined with PGE2/thrombin stimulation, drives the expression of inflammatory cytokines associated with LHX9. Further studies are needed to elucidate the molecular mechanisms governing the interactions between SERPINA1, PGE2/thrombin, and these DNA‐binding factors. Such research may facilitate the discovery of novel therapeutic targets for endometriosis.

## Author Contributions

**Kazuya Kusama:** conceptualization, methodology, data curation, investigation, validation, formal analysis, funding acquisition, visualization, writing – original draft, writing – review and editing, project administration. **Misuzu Ashihara:** investigation, validation, writing – review and editing. **Moana Okita:** investigation, validation, writing – review and editing. **Kanoko Yoshida:** investigation, validation, visualization, writing – review and editing. **Masumi Suzuki:** investigation, validation, writing – review and editing. **Kaito Suzuki:** validation, investigation, writing – review and editing. **Rena Hosokawa:** writing – review and editing, investigation, validation. **Mikihiro Yoshie:** writing – review and editing, supervision, validation, methodology. **Junya Kojima:** resources, investigation, writing – review and editing. **Yumi Mizuno:** investigation, resources, writing – review and editing. **Masanori Ono:** writing – review and editing, investigation, resources. **Hirotaka Nishi:** investigation, writing – review and editing, resources. **Takeshi Kajihara:** investigation, writing – review and editing, resources. **Kazuhiro Tamura:** conceptualization, data curation, supervision, writing – review and editing, writing – original draft, funding acquisition.

## Disclosure

The authors have nothing to report.

## Data Availability

The data sets used and/or analyzed during the current study available from the corresponding author on reasonable request. Primary sequence data were deposited in the DDBJ (https://www.ddbj.nig.ac.jp/dra/index-e.html; accession numbers: DRR304262 to DRR304273).
